# Quantized spin Hall conductance in a magnetically doped two dimensional topological insulator

**DOI:** 10.1038/s41467-021-23262-1

**Published:** 2021-05-27

**Authors:** Saquib Shamim, Wouter Beugeling, Pragya Shekhar, Kalle Bendias, Lukas Lunczer, Johannes Kleinlein, Hartmut Buhmann, Laurens W. Molenkamp

**Affiliations:** 1 Experimentelle Physik III, Physikalisches Institut, Universität Würzburg, Am Hubland, Würzburg, Germany; 2 Institute for Topological Insulators, Universität Würzburg, Am Hubland, Würzburg, Germany

**Keywords:** Quantum Hall, Topological insulators

## Abstract

Soon after the discovery of the quantum spin Hall effect, it has been predicted that a magnetic impurity in the presence of strong Coulomb interactions will destroy the quantum spin Hall effect. However, the fate of the quantum spin Hall effect in the presence of magnetic impurities has not yet been experimentally investigated. Here, we report the successful experimental demonstration of a quantized spin Hall resistance in HgTe quantum wells dilutely alloyed with magnetic Mn atoms. These quantum wells exhibit an inverted band structure that is very similar to that of the undoped material. Micron sized devices of (Hg,Mn)Te quantum well (in the topological phase) show a quantized spin Hall resistance of *h*/2*e*^2^ at low temperatures and zero magnetic field. At finite temperatures, we observe signatures of the Kondo effect due to interaction between the helical edge channels and magnetic impurities. Our work lays the foundation for future investigations of magnetically doped quantum spin Hall materials towards the realization of chiral Majorana fermions.

## Abstract (web summary)

The quantum spin Hall effect is expected not to survive the presence of magnetic impurities. Here, authors report full quantization at very low temperatures in HgTe quantum wells alloyed with a few percent of magnetic Mn atoms, due to Kondo screening.

## Introduction

The quantized spin Hall conductance is a hallmark of two dimensional (2D) topological insulators (TIs) where the conductance is solely due to a pair of counter propagating helical edge channels^1–3^. The stability of the quantum spin Hall (QSH) state in the presence of impurities has been the subject of numerous theoretical investigations^4–18^. For nonmagnetic impurities, which do not break time-reversal symmetry, the QSH states are robust against elastic single-particle backscattering events. Multi-particle scattering processes can, however, cause backscattering and hence lead to a suppression of the conductance away from the quantized value. Deviations from the quantized conductance of 2*e*^2^/*h* have been observed and attributed to various mechanisms including electron–electron interactions^8^, the presence of short range nonmagnetic impurities^17^ and tunneling to charge puddles^9,12^.

Magnetic impurities, on the other hand, can break time-reversal symmetry, and hence gap the QSH states. Maciejko et al. predicted that a magnetic impurity acting through the Kondo effect can lead to several interesting scenarios, depending on the strength of Coulomb interactions in the helical liquid^6^. For strong Coulomb interactions, two-particle backscattering processes^6^ lead to an insulating state at temperature *T* = 0. For weak Coulomb interactions, the magnetic impurity causes spin-flip scattering when *T* ≫ *T*_K_, where *T*_K_ is the Kondo temperature, resulting in a logarithmic decrease of conductance with decreasing temperature. Upon decreasing the temperature, at *T* ≪ *T*_K_ the conductance increases as a power law towards the quantized zero-temperature value 2*e*^2^/*h*^6^. Tanaka et al. show that the dc conductance is quantized to 2*e*^2^/*h* at all temperatures for isotropic Kondo interactions^7^. Anisotropic coupling of the spins of edge-state electrons to a macroscopic ensemble of magnetic impurities can lead to the spontaneous spin polarization of the impurities, resulting in the Anderson localization of edge states at *T* = 0^11^. Thus, there exist various theoretical predictions concerning the effect of magnetic impurities on the quantized conductance of a QSH insulator. Recent scanning tunneling microscopy experiments on one-dimensional topological edge states of bismuth, coupled to iron clusters, showed evidence of backscattering due to breakdown of time-reversal symmetry^19^. Such experiments, however, neither directly indicate the existence of a Kondo effect nor do they quantify the actual effect on the conductance quantization.

In spite of several theoretical predictions concerning the stability of the QSH effect, experimental investigations to test these predictions are rare, partly due to the limited number of available model systems, which demonstrate the quantized spin Hall conductance. A stable quantized plateau of 2*e*^2^/*h* has been successfully measured in transport experiments on nonmagnetic TIs based on HgTe quantum wells^3^, InAs/GaSb type-II quantum wells^20^ and monolayer WTe_2_^21^. Among the known quantum spin Hall systems, HgTe quantum wells provide an ideal platform to explore the rich phenomenology of the Kondo effect in magnetically doped TIs. This is because for dilute concentrations of Mn, the isoelectric substitution of Mn in HgTe causes neither additional carrier doping nor degradation in mobility. The weak Coulomb interactions in HgTe quantum wells^6^ suggest that (Hg,Mn)Te quantum wells are in the regime where Kondo physics due to the interaction of impurity spins with the helical edge channels can be investigated. A quantized spin Hall conductance in magnetically doped 2D TIs has, however, not yet been reported.

In this work, we report the observation of the quantum spin Hall effect in HgTe quantum wells with dilute concentration of Mn atoms. The longitudinal resistance is quantized to *h*/2*e*^2^ at zero magnetic field. Temperature dependent measurements show that the quantized conductance is stable from 18 to 400 mK. At higher temperatures, the conductance decreases with increasing temperature, in agreement with ref. ^6^, and is thus a signature of the Kondo effect due to interaction of helical edge channels and the spin of the magnetic impurity.

## Results and discussion

### Band structure calculated using the ${\mathbf{k}}$ ⋅ ${\mathbf{p}}$ method

**Fig. 1:**
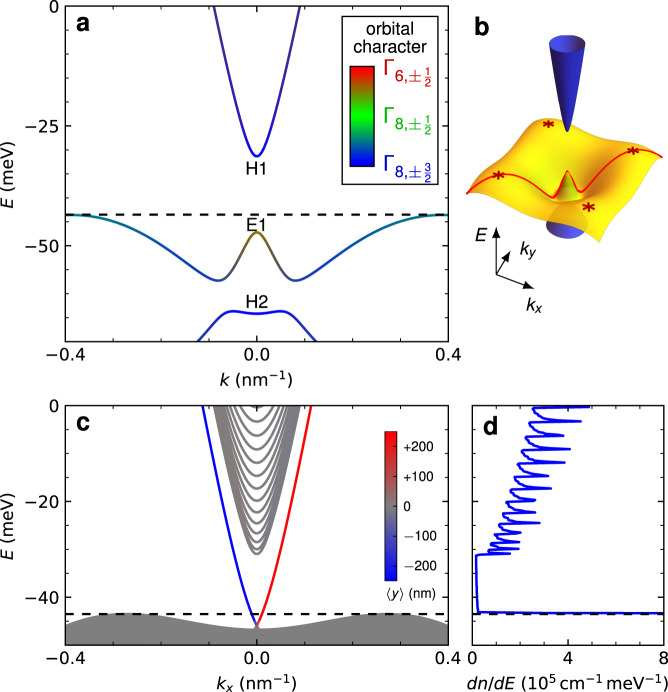
Band structures calculated with the ${\bf{k}} \cdot {\bf{p}}$ method. **a** Dispersion for an infinite (Hg,Mn)Te quantum well, 9 nm thick and with 1.2% Mn, along the axis $({k}_{x},{k}_{y})=k(\cos 4{5}^{\circ },\sin 4{5}^{\circ })=k(1/\sqrt{2},1/\sqrt{2})$. The colors indicate the orbital character, see legend. **b** Three-dimensional rendering of the band structure. We highlight the axis of part **a**, (red curve) and the camel back points (stars). **c** Dispersion in the strip geometry, 500 nm wide. The colors indicate the eigenstate location 〈*y*〉, see legend. **d** Density of carriers *dn*/*dE* for the strip geometry. Just below the camel back (*E* ≈ −46 meV, dashed lines), the density reaches values > 400 × 10^5^ cm^−1^ meV^−1^, out of scale in this plot.

In order to realize the QSH effect in a magnetically doped TI, we use a (Hg,Mn)Te quantum well that is 9 nm thick, with a Mn concentration of 1.2%. The energy *E* versus momentum *k* dispersion relation for this quantum well is calculated using an 8-orbital **k** ⋅ **p** method on a lattice for two scenarios: an infinite quantum well and a strip geometry^22,23^. In this model, we assume that the carriers couple to the average magnetization of the Mn ions (since their wave function covers a large number of Mn atoms), which responds paramagnetically to the external field at this concentration^22,24^. In absence of an external field, time-reversal symmetry is thus broken only at a microscopic level. Figure 1a shows the dispersion relation for an infinite (Hg,Mn)Te quantum well at zero-temperature and zero-magnetic field. We show the three subbands nearest to the gap, E1 with mixed ${{{\Gamma }}}_{6,\!\pm \frac{1}{2}}$ and ${{{\Gamma }}}_{8,\!\pm \frac{1}{2}}$ orbital character, and H1 and H2 with ${{{\Gamma }}}_{8,\!\pm \frac{3}{2}}$ orbital character, all of which are doubly degenerate. Due to the combination of the inverted gap of HgTe (${{{\Gamma }}}_{8,\!\pm \frac{3}{2}}$ states at higher energy than the ${{{\Gamma }}}_{6,\!\pm \frac{1}{2}}$ states) and strain effects, the subbands are also inverted, with the H1 subband above the E1 subband. This band ordering, similar to that of undoped HgTe quantum wells in ref. ^3^, implies the existence of a topological phase for a quantum well of the given thickness and concentration. The valence band (E1) shows maxima at finite momenta, which we refer to as the “camel back” feature. This feature is induced by a combination of the band inversion and hybridization between the subbands. The quantum well has an indirect band gap since the energy at the camel back is higher than the energy at *k* = 0. From the band structure as function of (*k*_*x*_, *k*_*y*_), shown in Fig. 1b, we find that the valence band maximum is reached at four points $k(\!\pm\! 1/\sqrt{2},\pm\! 1/\sqrt{2})$, at the diagonal lines between the *k*_*x*_ and *k*_*y*_ axes. The dispersion of Fig. 1a is plotted along the line $({k}_{x},{k}_{y})=k(\cos 4{5}^{\circ },\sin 4{5}^{\circ })=k(1/\sqrt{2},1/\sqrt{2})$ so that the camel back shown in this figure represents the true maximum of the valence band.

Additionally, we have performed **k** ⋅ **p** calculations on a strip of width 500 nm, having infinite length along the *x*-axis and spanning the range *y* = ±250 nm, in order to obtain the dispersions of the edge states. Figure 1c shows the band structure of the strip at zero magnetic field. The colors denote the location of the states along the width of the strip (see legend). One can clearly distinguish the edge states (red and blue) from the bulk states (gray). The Dirac point, where the edge states cross, is located just above the valence band at *k* = 0.

### Quantized spin Hall conductance in (Hg,Mn)Te quantum wells

**Fig. 2:**
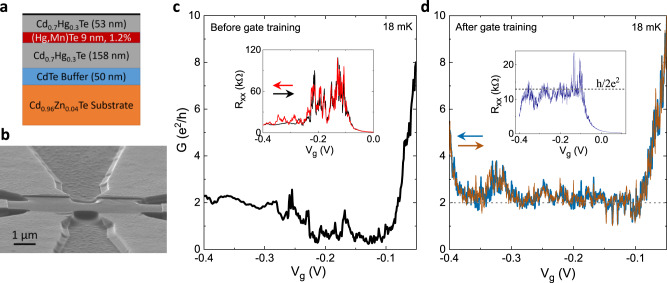
Quantized spin Hall conductance in a (Hg,Mn)Te quantum well. **a** Schematic of the layer stack showing the (Hg,Mn)Te quantum well sandwiched between two Cd_0.7_Hg_0.3_Te barriers on a Cd_0.96_Zn_0.04_Te substrate. **b** Scanning electron micrograph of a typical Hall bar used for measurements. **c** The conductance *G* as a function of gate voltage *V*_*g*_ for a 9 nm thick (Hg,Mn)Te quantum well with 1.2% Mn at 18 mK. The inset shows the longitudinal resistance *R*_*xx*_ as a function of *V*_*g*_. **d** *G* as a function of *V*_*g*_ at 18 mK after gate training. The inset shows *R*_*xx*_ as a function of *V*_*g*_. The dotted line indicates the expected conductance of 2*e*^2^/*h* in the quantum spin Hall regime. The arrows in **c** and **d** show the direction of gate voltage sweeps.

The (Hg,Mn)Te quantum well used in this experiment has been grown by molecular beam epitaxy on lattice-matched Cd_0.96_Zn_0.04_Te substrate that provides minimal strain to the layers. The (Hg,Mn)Te quantum well is embedded between Hg_0.3_Cd_0.7_Te barriers with thicknesses of 53 nm (top barrier) and 158 nm (bottom barrier). A schematic of the layer stack is shown in Fig. 2a. The electron mobility for this quantum well is ~2 × 10^5^ cm^2^V^−1^s^−1^ at a carrier density of *n* ~ 5 × 10^11^ cm^−2^ as estimated from magneto-transport measurements in low perpendicular magnetic field.

Previous investigations on HgTe quantum wells have shown that the QSH effect is most easily observed in micron-sized Hall bars. We use a chemical wet-etching process to fabricate high mobility QSH microstructures in a six-terminal Hall bar geometry of dimensions 1.9 × 1.7 μm^2^, with the same crystal quality as the original MBE grown material^25^. A 14 nm thick HfO_2_ layer grown by atomic layer deposition is used as a dielectric insulator. The carrier density can be controlled by applying a voltage to a 5/200 nm Ti/Au gate layer deposited on the HfO_2_ insulator. The ohmic contacts are formed by depositing 50 nm of AuGe and 50 nm of Au by e-beam evaporation. More details of the device fabrication process can be found in ref. ^25^. A scanning electron micrograph of a typical micro Hall bar (without the top gate) is shown in Fig. 2b.

All electrical transport measurements have been performed in a dilution refrigerator at a base temperature of 18 mK. The resistance of the device has been measured in a four terminal configuration using low frequency (≈13 Hz) lock-in techniques. The device can be tuned from *n*-type conduction for *V*_*g*_ ≥ 0 V to *p*-type conduction for *V*_*g*_ ≤ −0.4 V (Fig. 2c). The bulk band gap regime is identified by an increased resistance (or decreased conductance in Fig. 2c) for *V*_*g*_ = −0.1 to −0.2 V (inset of Fig. 2c). We note that in the bulk gap regime *R*_*xx*_ is much larger than the expected quantized resistance of *h*/2*e*^2^. We have recently shown that such a deviation from the quantized value results from charge puddle induced potential fluctuations^26^. In the present sample, the resistance (when the chemical potential is in the bulk band gap) shows large fluctuations (~tens of kΩ) for the virgin gate sweep, which is a characteristic of our Mn-doped HgTe devices. For undoped HgTe structures, the fluctuations in the quantized spin Hall resistance are much smaller ~1 kΩ^3,25^. The measured fluctuations in *R*_*xx*_ are observed only in microscopic devices as they are due to scattering from a small number of charge puddles. As has been shown in ref. ^26^, the average distance between puddles is ~few *μ*m, which implies that a micron-sized device is affected by only a few charge puddles. A comprehensive analysis of the conductance fluctuations and their dependence on temperature will be discussed in a future publication.

Reference^26^ also demonstrates that in our HgTe samples it is possible to smooth out the potential landscape and thus approach the expected QSH resistance by repeated gate sweeps (“gate training”). We follow a similar procedure and sweep the gate voltage multiple times (~300) between 0.1 and −0.4 V, the range for which we do not observe any gate hysteresis. The gate voltage characteristics of the device at 18 mK after gate training show an excellent quantization of the conductance *G* to 2*e*^2^/*h* for a range of *V*_*g*_ from −0.1 V to −0.3 V (Fig. 2d). The inset of Fig. 2d shows the quantization in resistance as a function of *V*_*g*_ at 18 mK. Apart from the conductance quantization, another noticeable effect of gate training is that the fluctuations in resistance are now of the order ~kΩ (inset of Fig. 2d), an order of magnitude smaller as compared to the device before gate training. The observation of a quantized conductance and reduced fluctuations after gate training is clear evidence that we have achieved a smoother potential landscape. The quantized spin Hall conductance in a magnetically doped 2D TI at zero magnetic field clearly indicates that the edge channels are robust also in the presence of magnetic impurities. This is the key observation of the paper. The quantized conductance due to quantum spin Hall effect has been reproduced in this device after a thermal cycle and in other devices fabricated from the same wafer. Devices with different Mn concentrations (<2.5%) have also shown the conductance quantization due to the quantum spin Hall effect.

### Temperature dependence of the quantized spin Hall conductance: signature of the Kondo effect

**Fig. 3:**
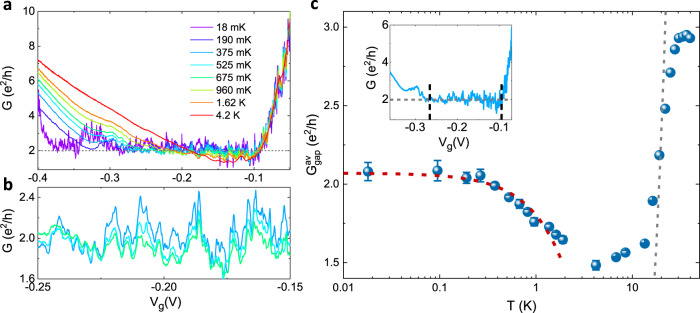
Effect of temperature on the quantized conductance. **a** The conductance *G* as a function of gate voltage *V*_*g*_ at different temperatures from 18 mK to 4.2 K. The dotted line indicates the expected conductance of 2*e*^2^/*h* in the quantum spin Hall regime. **b** Magnified view of *G* as a function *V*_*g*_ for different temperatures to show the reproducibility in conductance fluctuations. **c** The average conductance in the quantum spin Hall regime ${G}_{{\rm{gap}}}^{{\rm{av}}}$ as a function of temperature *T*. The blue points are the experimental data and the red dashed line shows the power-law Kondo fit (see main text). The gray dashed line shows the expected thermally activated conductance [$\propto \exp (-{E}_{g}/2{k}_{B}T)$] for a band gap of *E*_*g*_ = 10 meV (from the band structure calculations shown in Fig. 1). The inset shows a typical *G*–*V*_*g*_ dependence from which ${G}_{{\rm{gap}}}^{{\rm{av}}}$ (shown by dashed horizontal line) is calculated by averaging over the conductance values in the plateau region between the dashed vertical lines.

To probe the temperature limit up to which the QSH effect persists in our magnetically doped TIs, we have performed transport measurements at various temperatures. The *G*–*V*_*g*_ traces at temperatures ranging from 18 mK to 4.2 K (Fig. 3a) show several distinct features. The fluctuations in conductance are reproducible at different temperatures (Fig. 3b) and hence are not an artefact of measurement noise. The fluctuations in conductance are largest at lowest temperature and decrease as the temperature increases. The average conductance in the QSH regime, ${G}_{{\rm{gap}}}^{{\rm{av}}}$, is obtained by averaging over the conductance values in the plateau region of the *G*–*V*_*g*_ curve (region between the vertical dashed lines in the inset of Fig. 3c, note that the length of the plateau region decreases at higher temperatures). The error in determining ${G}_{{\rm{gap}}}^{{\rm{av}}}$ at a particular temperature has been estimated by calculating the standard deviation of the ${G}_{{\rm{gap}}}^{{\rm{av}}}$ values obtained by choosing multiple (>5) realistic plateau regions of the *G*–*V*_*g*_ curve. The average conductance in the QSH regime is constant up to 400 mK and subsequently decreases with increasing temperature (blue points in Fig. 3c). This observation is qualitatively in agreement with ref. ^6^, where it is predicted that for weak Coulomb interactions, the magnetic impurity spin is fully screened by the edge electrons at *T* = 0 and the conductance is quantized to 2*e*^2^/*h*. An increase in temperature leads to a decrease in conductance because the impurity spin is not fully screened at higher temperatures and causes inelastic scattering. The observation of quantized conductance at low temperatures and subsequent decrease in conductance at higher temperature (>400 mK) is a manifestation of the Kondo effect due to interaction of helical edge channels with the magnetic impurities. In contrast, for HgTe quantum wells without Mn doping, we have observed quantized conductance up to 15 K^25^. While a spin glass phase due to antiferromagnetic ordering of Mn has been observed for II–VI compounds doped with high concentration of Mn (7–20%) at millikelvin temperatures^27^, which can cause scattering and fluctuations in conductance, we observe a quantized conductance from 18–400 mK and hence can rule out scattering due to antiferromagnetic ordering at low temperatures.

From refs. ^5,6^, for weak Coulomb interactions, the decrease of conductance with increasing temperature follows a power law $G={G}_{0}-\alpha {(T/{T}_{{\rm{K}}})}^{2(4K-1)}$, where *G*_0_ is the conductance due to quantized spin Hall edge channels, *α* is a proportionality constant and the interaction parameter *K* quantifies the strength of Coulomb interactions in the helical edge channels. For HgTe quantum wells, the Coulomb interactions are expected to be weak^28,29^. Hence we fit the low temperature part of the ${G}_{{\rm{gap}}}^{{\rm{av}}}(T)$ dependence with the expression $G={G}_{0}-\alpha {(T/{T}_{{\rm{K}}})}^{2(4K-1)}$ (red dashed curve in Fig. 3c is the fit) and obtain a reasonable value of *K* ~ 0.4. The proportionality constant *α* was set to *e*^2^/*h*, *G*_0_ = 2.07*e*^2^/*h* and *T*_K_ ~ 3 K. Since this is a multi-parameter fit for a rather limited range of temperature, we cannot conclusively rule out other mechanisms of temperature dependent corrections to the QSH conductance. However, samples without Mn doping do not show the pronounced decrease in conductance with increasing temperature in the range of 100 mK to a few K observed here. In Mn-free samples, the quantized conductance due to the quantum spin Hall effect is temperature independent up to *T* ~ 15 K^25^. Hence, the initial decrease in conductance in the sub-Kelvin range in Fig. 3c is a strong indication of the Kondo origin of this observation. An independent estimate of *K* can be obtained from the formula^28^,

1 $$K={\left[1+\frac{2}{\pi }\frac{{e}^{2}}{{\epsilon }_{0}{\epsilon }_{r}\hslash {v}_{F}}{\mathrm{ln}}\,\left(\frac{7.1d}{\xi +0.8w}\right)\right]}^{-1/2}$$

where *ϵ*_0_ is the permittivity of free space, *ϵ*_*r*_ is the dielectric constant, *v*_*F*_ is the Fermi velocity, *d* is the distance between the edge channel and the gate, *w* is the thickness of the quantum well, *ξ* = 2*ℏv*_*F*_/*E*_*gap*_ and *E*_*gap*_ is the band gap of the system. Using *ϵ*_*r*_ = 20 for HgTe, *v*_*F*_ = 5.5 × 10^5^ m s^−1^, *d* = 70 nm, *w* = 9 nm, and *E*_*gap*_ = 10 meV, we get *K* ~ 0.5, which is in good agreement with the value obtained by fitting the experimental data in Fig. 3c. For *T* > *T*_K_, the conductance increases slowly with increasing temperature (up to ~15 K) as expected for spin-flip scattering off a magnetic impurity^6^. Above 15 K, the conductance increases sharply with increasing temperature since the thermal excitations across the bulk band gap of 10 meV (from **k** ⋅ **p** band structure calculations shown in Fig. 1) strongly contribute to the measured conductance. To illustrate this, we show in Fig. 3c (gray dashed line), the expected thermally activated conductance [$\propto \exp (-{E}_{g}/2{k}_{B}T)$] for a band gap of *E*_*g*_ = 10 meV, which agrees with the experimentally observed behavior. Hence, for higher temperatures, the conductance increases above the expected quantized value. The saturation in conductance for *T* > 30 K is due to enhanced phonon scattering at high temperatures^30^.

In addition, we observe that the length of the quantized plateau (in *V*_*g*_) in Fig. 3a decreases strongly with increasing temperature. For the *G*–*V*_*g*_ curves shown in Fig. 3a, there is no “isosbestic point” (the curves for different temperatures do not cross at the same point). This suggests that the temperature dependence of conductance for large negative *V*_*g*_ is determined by processes, which are different from those described in the previous paragraph for the temperature dependence of ${G}_{{\rm{gap}}}^{{\rm{av}}}$. To understand the temperature dependence of the length of the quantized plateau and the conductance for large negative *V*_*g*_, we have calculated the density of states from the dispersion shown in Fig. 1c. There is an extremely large density of states in the valence band, arising from the camel back, which is ~50–100 times larger than that in the conduction band (Fig. 1d). This large density of states near the camel back causes ‘pinning’ of the chemical potential, i.e., the position of the chemical potential is almost unchanged for a large range of gate voltages. Even though the camel back (bulk) states are occupied, they do not contribute to the conductance at lowest temperatures (18 mK) as the carriers are localized by disorder^23^. Hence the conductance is quantized to the QSH value of 2*e*^2^/*h* for a large range of gate voltage at 18 mK (Fig. 2c). We have shown in ref. ^23^, that for higher temperatures the carriers in the camel back are delocalized. Hence, the measured longitudinal conductivity rises above 2*e*^2^/*h* from contributions of bulk states at the top of the camel back, resulting in a strong increase of conductance with increasing temperature for *V*_*g*_ < −0.3 V.

To summarize, we have performed transport measurements in a magnetically doped TI realized by incorporating Mn atoms in a HgTe quantum well. The conductance of the micron-sized Hall bar is quantized to 2*e*^2^/*h* up to 400 mK and subsequently decreases with increasing temperatures, confirming the prediction of interaction of helical edge channels with a Kondo impurity^6^. Our observation of quantized conductance in (Hg,Mn)Te quantum wells should lead to further investigations of the Kondo effect in magnetically doped 2D TIs, particularly in the presence of tunable Rashba interaction which can control the Kondo temperature^31^, thus making the Kondo crossover (the regime for *T* ≫ *T*_K_ where conductance increases with increasing temperature) accessible in the transport experiments. Transport investigations in devices patterned in Corbino geometry can give further insights of the edge-state transport in magnetically doped quantum spin Hall materials. The realization of quantized spin Hall conductance in a magnetically doped TI lays the groundwork for further experiments on Majorana fermions by interfacing these structures with superconductors.

## Data Availability

All data necessary to support the conclusions of the paper are available in the manuscript. Source data are provided with this paper.

## Source data

Source Data

## Code availability

The code used for the band structure analysis is available from the corresponding authors upon reasonable request.
